# Interindividual Variation in Source‐Specific Doses is a Determinant of Health Impacts of Combined Chemical Exposures

**DOI:** 10.1111/risa.13550

**Published:** 2020-07-15

**Authors:** Paul Price

**Affiliations:** ^1^ Office of Research and Development United States Environmental Protection Agency Washington DC USA

**Keywords:** Chemical risk assessment, combined exposures, simulation modeling

## Abstract

All individuals are exposed to multiple chemicals from multiple sources. These combined exposures are a concern because they may cause adverse effects that would not occur from an exposure recieved from any single source. Studies of combined chemical exposures, however, have found that the risks posed by such combined exposures are almost always driven by exposures from a few chemicals and sources and frequently by a single chemical from a single source. Here, a series of computer simulations of combined exposures are used to investigate when multiple sources of chemicals drive the largest risks in a population and when a single chemical from a single source is responsible for the largest risks. The analysis found that combined exposures drive the largest risks when the interindividual variation of source‐specific doses is small, moderate‐to‐high correlations occur between the source‐specific doses, and the number of sources affecting an individual varies across individuals. These findings can be used to identify sources with the greatest potential to cause combined exposures of concern.

## 1 INTRODUCTION

Individual human or ecological receptors’ exposure to chemicals are complex (Escher, Stapleton, & Schymanski, 2020; Paulik & Anderson, 2018). All individuals are exposed to large numbers of chemicals and an individual may have multiple sources of exposure to a single chemical. In the United States, an individual's exposures to a single chemical from multiple sources is referred to as aggregate exposure, exposures to multiple chemicals as cumulative exposures (Environmental Protection Agency, 2003), and in the United States and Europe both types have been referred to as combined exposures (Organisation for Economic Co‐operation and Development, 2018). The concern raised by such exposures is that while an individual may be able to tolerate a dose of one chemical from a single source, the combined doses of one or more chemicals recieved from multiple sources may result in adverse effects. As a result, findings of safety for of doses of individual chemicals received from a single source may not be protective of the health of humans or ecological receptors.

Risk assessors established screening methods for assessing risks from combined exposures that are based on dose‐addition models (Environmental Protection Agency, 1986; Escher et al., 2020; Stokinger, 1964). Combined exposure assessments for single chemicals sum source‐specific doses to arrive at estimates of total doses. Combined exposure assessments for multiple chemicals use chemical‐specific indices to convert the doses of different chemicals into equivalent doses of a toxicity‐weighted metric. These metrics are summed to give a measure of the combined toxicity of the chemicals to the individual.

As demonstrated in the Methods section, these additive risk models can be reduced to a single equation. Safety for an individual is assumed when the following is true:

(1) $$1\geq \sum_{k}^{m}\sum_{i}^{n}TD_{ijk},$$

where *TD_ijk_* is the toxicity‐weighted dose of the *i*th chemical for the *j*th individual from the *k*th source for *n* chemicals and *m* sources. A measure of combined toxicity for an individual (*CTD_j_*) is given by the sum of the toxicity‐weighted doses:

(2) $$CTD_{j}=\sum_{k}^{m}\sum_{i}^{n}TD_{ijk}.$$

The contribution of the chemical with the largest *TD_ijk_* to *CTD_j_* is characterized using the maximum cumulative ratio (MCR) (Price & Han, 2011). The MCR is a property of the individual and is defined as:

(3) $$MCR_{j}=CTD_{j}/\mathrm{Max}(TD_{ij}).$$

Values of *MCR_j_* range from one to *n*. *MCR_j_* values approaching one indicate that the contribution of one chemical dominates *CTD_j_* and values approaching *n* indicate that the chemicals are present in equitoxic doses (Price & Han 2011). For example, an individual having toxicity‐weighted doses from three chemicals of 0.10, 0.10, and 0.11 and a second individual having 0.10, 0.10, and 2.0 would have MCR values of 2.8 and 1.05.

**Fig 1:**
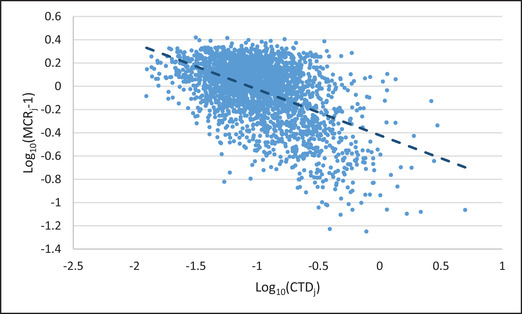
Plot of log_10_(*MCR_j_* − 1) versus log_10_(*CTD_j_*) showing MCR slope for a population of approximately 2,000 individuals with exposures to six phthalates (Reyes & Price, 2018b).

Studies of interindividual variation in *MCR_j_* have been published for populations of humans and ecological receptors. Studies have been performed on exposures to sources such as mixtures of chemicals in ground and surface water (Han & Price, 2011; Holmes et al., 2018; Price & Han, 2011; Price et al., 2012; Silva & Cerejeira, 2015; Vallotton & Price, 2016), in indoor air (De Brouwere et al., 2014; Mishra, Ayoko, Salthammer, & Morawska, 2015), and cumulative exposures predicted from data collected in biomonitoring studies (Han & Price, 2013, Reyes & Price, 2018a, 2018b). The studies show a consistent pattern for *MCR_j_* in the study populations. Values of *MCR_j_* vary across individuals but values are typically closer to one than *n*. Even when the data include findings on large numbers of chemicals (*n* of 20 or more), values of *MCR_j_* are typically less than five and average less than two. In addition, *MCR_j_* is consistently found to be negatively correlated with *CTD_j_*. This correlation is measured by taking the slope of the log(*MCR_j_* – 1) versus the log(*CTD_j_*) and is hereafter referred to as the MCR slope (Reyes and Price, 2018b). Fig. 1 presents an example of such a slope for estimates of combined exposures to six phthalates in U.S. adults and children as measured by biomonitoring.

This pattern has significant implications for predicting risks posed by combined exposures. The negative values of the MCR slope results in the most exposed individuals having MCR values close to one. When this occurs, the upper bounds of the populations’ combined exposures are similar to the upper bounds of the largest of the individual source‐specific doses. This implies that if the upper bounds of the doses from the sources of exposure for these populations individually do not pose risk, then they are not likely to pose a risk in combination. In populations where the pattern occurs, individuals are not likely to have a risk that would be missed by not performing a combined exposure assessment. If criteria are identified that predict when the pattern does, and does not occur, then the criteria can be used to focus combined exposure assessments on populations that are the greatest concern.

This article explores whether this pattern occurs as the result of the shapes of the distributions of source‐specific values of *TD* across individuals in exposed populations. It is difficult to find an analytical solution to the relationship between the shapes of the source‐specific distributions of TD on the distribution of *MCR* values across a population. The relationship is therefore investigated empirically by using a simulation model of interindividual variation in individuals’ combined exposure in a population exposed to chemicals from multiple sources.

## 2 MATERIALS AND METHODS

This section is divided into three sections. The first section is a demonstration that many of the existing approaches used to assess combined exposures involving one or more chemicals reduce to a common formula. This formula is used as the basis for the design of the simulation model. The second section presents a description of the simulation model. The final section describes how the model is used to investigate the impacts that the characteristics of interindividual variation in the source‐specific values of *TD* have on MCR values.

### 2.1 Derivation of a Common Model for Dose‐Additive Aggregate and Cumulative Risk Assessments

Both aggregate and cumulative risk assessments address the issue that an individual's chemical exposures occur as a result of the individual's interactions with multiple sources of exposure that result in multiple doses of one chemical (for aggregate assessments) or two or more chemicals (for cumulative assessments). In both types of combined assessments, a dose metric is determined for each source of exposure. The source‐specific dose metrics are summed across the sources for aggregate assessments and across both chemicals and sources for cumulative assessments. The resulting sums are evaluated against a criterion derived from toxicity data.

For aggregate exposures, the dose metric is the source‐specific dose of a chemical. These are summed over all sources to give the aggregate dose of the chemical to the individual:

(4) $$AD_{ij}=\sum_{k}^{m}D_{ijk}\;,$$

where $D_{ijk}$ is the dose of the *i*th chemical for the *j*th individual from the *k*th source, *m* is the number of sources, and *AD_ij_* is the aggregate dose to the *i*th chemical to the *j*th individual. The risk is judged to be acceptable when the aggregate dose is less than the permitted dose of the *i*th chemical (*PD_i_*):

(5) $$AD_{ij}\leq PD_{i}.$$

For cumulative risk assessments, the values of *D_ijk_* are determined separately for each chemical and source. The doses are then normalized to a common metric that allows their summation. Two commonly used methods for normalization are the hazard index (HI) (Environmental Protection Agency, 1986) and toxicity equivalents (TEqs) (Varshavsky, Morello‐Frosch, Woodruff, & Zota, 2018).

The HI method normalizes *D_ijk_* by dividing *D_ijk_* by the permitted dose of the *i*th chemical (*PD_i_*) to give the hazard quotient metric (*HQ_ijk_*):

(6) $$HQ_{ijk}=D_{ijk}/PD_{i}.$$

An individual's *HQ_ijk_* values are summed over *n* chemicals and *m* sources to give the individual's HI (*HI_j_*):

(7) $$HI_{j}=\sum_{k}^{m}\sum_{i}^{n}HQ_{ijk}.$$

The risk from cumulative exposures is judged to be acceptable for the *j*th individual when the value of *HI_j_* is less than 1.

The TEq method normalizes *D_ijk_* by multiplying it with a chemical‐specific toxicity equivalent factor (*TEF_i_*) that converts the chemical‐specific dose to equivalent dose of an index chemical (*ID_ijk_*):

(8) $$ID_{ijk}=D_{ijk}*TEF_{i}.$$

These equivalent doses are summed over *n* chemicals and *m* sources to produce an estimate of the total dose of the index chemical reaching the *j*th individual (*ID_j_*):

(9) $$ID_{j}=\sum_{k}^{m}\sum_{i}^{n}ID_{ijk}.$$

When the value of *ID_j_* is less than the permitted dose for the index chemical (*PD_ID_*), the individual's risk from cumulative exposures is judged to be acceptable.

The finding of acceptability of an individual's combined exposures as measured by the aggregate and two cumulative methods can be fit to a single equation. An individual's combined exposures is acceptable when the following is true:

(10) $$1\geq \sum_{k}^{m}\sum_{i}^{n}K_{i}D_{ijk}.$$

The value of *K_i_* is 1/*PD_i_* for aggregate assessments and cumulative assessment that use the HI approach and *TEF_i_/PD_ID_* for cumulative assessments that use the TEq approach. If *K_i_D_ijk_* is defined as the toxicity‐adjusted dose of the *i*th chemical for the *j*th individual from the *k*th source (*TD_ijk_*), then all three methods reduce to:

(11) $$1\geq \sum_{k}^{m}\sum_{i}^{n}TD_{ijk}.$$

The measure of combined toxicity adjusted doses for the *j*th individual (*CTD_j_*) is defined as:

(12) $$CTD_{j}=\sum_{k}^{m}\sum_{i}^{n}TD_{ijk}.$$

Equations (11) and (12) do not address the issue of route of exposure on dose. In an actual cumulative or aggregate risk assessment, route‐specific adsorption and metabolism would need to be assessed to determine how route‐specific doses would contribute to the total systemic dose of the individual. In addition, the interindividual variation in *TD_ijk_* for aggregate assessments differs from the variation in cumulative assessments. In aggregate assessments, variation in *TD_ijk_* is only a function of variation in *D_ijk_* since *K_i_* is a constant. For the two cumulative models, the interindividual variation in *TD_ijk_* is a function of both the variation in *D_ijk_* and *K_i_*.

The value of the MCR for the *j*th individual can be defined as:

(13) $$MCR_{j}=CTD_{i}/\mathrm{Max}\left(TD_{ij}\right).$$

In this article, the term MCR is also used to refer to the corresponding ratio in aggregate exposure assessments (aggregate dose divided by the single largest source‐specific dose):

(14) $$\;MCR_{j}=\frac{\sum_{k}^{m}D_{jk}}{\mathrm{Max}\left(D_{jk}\right)}\;.$$

While such a ratio would be, strictly speaking, the maximum aggregate ratio rather than a MCR, it behaves in the same way as the MCR and can be investigated using the same approaches.

### 2.2 Design of the Simulation Model

The design of the model is straightforward. A series of *m* sources of chemical exposure are characterized, and toxicity‐adjusted doses received from each source by each individual in an exposed population are determined. For simplicity, the model assumes that each source releases a single unique chemical. As a result, the number of sources and chemicals are the same (*m* equals *n*), the maximum number of *TD* for an individual is *m*, and the variation in *TD* values across sources reflects differences between both the exposure potentials of the sources and the chemicals' toxicities. No attempt is made to separate the contribution of the two sources of variation. Values of *CTD_j_* and $MCR_{j}$ are determined for each individual using the values of *TD_jk_*. This process is repeated for a large number of populations in each model run and the characteristics of the distributions of the source‐specific doses are varied across the populations. Three metrics are tracked for each population; the median value of $MCR_{j}$ in the population (median MCR), the average value of $MCR_{j}$ for the individuals with the 10 largest values of *CTD_j_* in the population (high‐risk MCR), and the MCR slope.

The model characterizes three types of variation. At the most basic level, the model needs to characterize variation in toxicity weighted doses received from a source across the individuals in the simulated population (interindividual variation in *TD_jk_*). Second, the model needs to characterize variation in the distributions of source‐specific doses across the *m* sources of exposures of a population (intrapopulation variation in the distributions of *TD_jk_*). Finally, the model must characterize the differences in the ranges of the *m* distributions in the different simulated populations (interpopulation variation of interpopulation variation in the distributions of *TD_jk_*).

The values of *TD_jk_* differ across the individuals in a population as a function of the level of interaction each individual has to each source and the physical and chemical properties of the chemicals that influence intake and uptake of the substances. In the simulation modeling, the interindividual variation of *TD_jk_* are modeled using lognormal distributions. Distributions of source‐specific doses are bounded at zero, frequently vary over several orders of magnitude, and tend to be right skewed. The distributions of such doses have been shown to be well approximated by lognormal distributions (Hattis & Burmaster, 1994; Limpert, Stahel, & Abbt, 2001; Ott, 1978). This occurs in part, because concentrations of chemicals in environmental media tend to follow lognormal distributions (Ott, 1990; Rappaport & Kupper, 2008). Dose estimates, expressed in units of mass per body weight, are also the product of multiple factors (bodyweight, duration of exposure, intensity of exposure, and uptake rates) that are multiplied or divided by one another. Such multiplicative formulae also tend to produce lognormally distributed results (Limpert et al., 2001).

**Fig 2:**
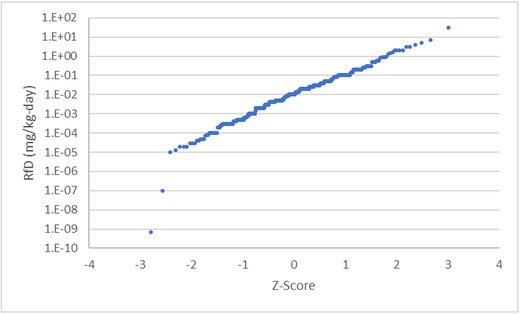
Q–Q plot of noncancer oral reference dose values for 381 substances.

For combined assessments involving multiple chemicals, the value of *K_i_* also varies across chemicals. As a result, the chemical‐specific distributions of *TD_jk_* across the individuals in a population will have the variation in their averages increased as a result of variation in *K_i_*. Interchemical variation in *toxicity* values also follow right‐skewed distributions, are bounded at zero, and vary by more than six orders of magnitude. For example, Fig. 2 shows a *Q*–*Q* plot of the 381 reference doses (a type of permitted dose) reported on the U.S. Environmental Protection Agency's integrated risk information system^1^. Except for the two most toxic compounds, the data are linear indicating the values follow a lognormal distribution.

Populations exposed to multiple sources include individuals who are exposed to some but not all sources. For example, an exposed population could include hobbyists who use the chemical as part of their hobbies and nonhobbyists who do not. The distribution of *TD_jk_* should therefore assign values of zero for *TD_jk_* for some individuals and sources. This requires a mixed distribution where a parametric distribution describes variability in *TD_jk_* for a portion of the population exposed to the *k*th source ($\alpha_{k}$) and a dose of zero is assigned to the remaining portion (1 − $\alpha_{k}$). The final distribution from the *k*th source is described using three inputs, a geometric mean (*GM_k_*), a geometric standard deviation (*GSD_k_*), and the fraction of the population that is exposed ($\alpha_{k}$):

(15) $$\begin{array}{ccc} & & \;\;\;\;\;TD_{ijk\;}\left(\alpha_{ik},GM_{ik},GSD_{ik}\right)∼\\ & & \;\left\{\begin{array}{c} \;\mathrm{Lo}g_{10}\mathrm{normal}\;\left(GM_{ik},GSD_{ik}\right)\;,\;\hat{\alpha }<\alpha_{ik}\\ 0,\;\;\;\;\;\;\;\;\;\hat{\alpha }\geq \alpha_{ik} \end{array}\right\}.\; \end{array}$$

Different sources of exposure produce different distributions of doses. Because of these differences, the values of the three inputs vary across sources. The goal in the selection of the distributions of the values of the three inputs is to evenly sample the parameter space of the inputs. As a result, uniform or log‐uniform distributions are used to describe variation in the source‐specific values of the three inputs. In addition, in order to investigate the impact of differences in the values of *GM_k_*, *GSD_k_*, and $\alpha_{k}$, the various populations need to reflect different ranges of values for these inputs. Therefore, the averages and ranges of the inputs are also varied in a uniform way across the populations. The intrapopulation and interpopulation variability are modeled separately for *GM_k_*, *GSD_k_*, and $\alpha_{k}$ using the following approaches.

The values of the daily doses of a chemical that an individual receives from a source could range from a few molecules to several hundred grams. This suggests that in the simulation models, the values of *GM_k_* and *GSD_k_* should be allowed to result in a wide range of values for *TD_jk_*. The average of *GM_k_* for the sources in a population is a scaler that equally effects all values of *GM_k_* and by extension all values of *TD_jk_* and *CTD_j_*. Since $MCR_{j}$ is the ratio of *CTD_j_* and max(*TD_jk_*), the values of the three metrics (median MCR, high‐risk MCR, and MCR slope) are all independent of the average of *GM_k_*. While the average value of *GM_k_* has no impact on the three metrics, the range in the source‐specific values of *GM_k_* for the *m* sources of a population does have an effect. The intrapopulation variation in the spread of values of *GM_k_* is modeled by assuming that the values of *GM_k_* in a population's sources follow a log_10_ uniform distribution. The range can be described by assigning a value of one for the average value of *GM_k_* and using a spread parameter (*S_GM_*) that is defined as follows:

(16) $$S_{GM}=\mathrm{lo}g_{10}{\left(GM_{k}\right)}_{\max}-\mathrm{lo}g_{10}{\left(GM_{k}\right)}_{\min}.$$

The maximum and minimum values of the logs of the averages of the source‐specific dose distribution for a population, log_10_(*GM_k_*)_min_ and log_10_(*GM_k_*)_max_, are then 0.5 *S*_GM_ and −0.5 *S*_GM_. When *S_GM_* is equal to zero for a population, the *GM_k_* of all sources has a value of one. A value of six indicates that the values of *GM_k_* for the sources of a population could vary by a factor of 1 million. Modeling interpopulation differences in the spread of *GM_k_* is performed by varying the value of *S_GM_* across the sources. The interpopulation variation in *S_GM_* is modeled by sampling from a uniform distribution between zero and the maximum population spread (*PS_GM_*) in the simulation run.

The intrapopulation variation in *GSD_k_* across sources also can be large. Values of *TD_jk_* from a source can vary over the population's individuals by many orders of magnitude due to differences in chemical concentrations in relevant media and variation in the rate of intake and uptake of the chemicals. For another source, where the concentrations are fixed and exposures occur in a similar way, the source‐specific doses may be relatively constant across the population. *GSD_k_* is also limited to values greater than one. The average value of *GSD_k_* as well as the range affects the values of the three population metrics. Therefore, both the inter‐ and intrapopulation variation of the average and the spread of *GSD_k_* need to be considered in the model.

Simulating the values of *GSD_k_* for a population's sources uses an approach similar to that used for *GM_k_*. The intrapopulation variation in *GSD_k_* is again modeled using a log_10_ uniform distribution defined by log_10_(*GSD_k_*)_min_ and log_10_(*GSD_k_*)_max_. The values of log_10_(*GSD_k_*)_min_ and log_10_(*GSD_k_*)_max_ are defined using the average and spread of the values of $\mathrm{lo}g_{10}\left(GSD_{k}\right)$ in a population's sources (*M_GSD_* and *S_GSD_*). *S_GSD_* is defined as:

(17) $$S_{GSD}=\mathrm{lo}g_{10}{\left(GSD_{k}\right)}_{\max}-\mathrm{lo}g_{10}{\left(GSD_{k}\right)}_{\min}.$$

The values of log_10_(*GSD_k_*)_min_ and log_10_(*GSD_k_*)_max_ are then *M_GSD_* plus or minus 0.5 × *S_GSD_*. The interpopulation variation in *M_GSD_* is determined by sampling values of *M_GSD_* from a uniform distribution of between 0 and a maximum value of *M_GSD_* for all populations in a simulation run (*PM_GSD_*). Because values of log_10_(*GSD_k_*)_min_ must be greater than zero, the value of *S_GSD_* for a population must be less than or equal to 2 × *M_GSD._* The intrapopulation variation in *S_GSD_* is therefore sampled from a uniform distribution between zero and 2 × *M_GSD_*.

Values for $\alpha_{k}$ fall between 0 and 1. In the simulation model, the intrapopulation variation in $\alpha_{k}$ is modeled as uniform distributions that vary from *α_k‐_*_min_ to *α_k‐_*_max_. In this model, values of *α_k‐_*_min_ and *α_k‐_*_max_ are held constant across the populations in a model run (interpopulation variation is zero).

An individual's exposures to one source may be correlated to exposures of a second. The simulation model is used to investigate the impacts of correlation of *TD* between sources. In this analysis, correlation is modeled using rank correlation. All sources are assumed to be similarly correlated. This assumption exaggerates the impacts of correlation since in many instances different levels of correlation occur between different pairs of sources.

The analyses were performed in Excel^TM^ using the Excel add on software @Risk^TM^ (version 7.6.0). A copy of the spreadsheet containing the model used in this analysis is provided in the Supporting Information.

### 2.3 Model Runs and Outputs

Six model runs of 5,000 populations each containing 1,000 individuals were performed (Table I). The values of *m* for the first and second runs were held constant at 20 and 100 respectively. Each individual was assumed to be exposed to all sources. The third run had an *m* of 100, but each individual had a 20% chance of exposure to each source. In this run, the average individual was exposed to 20 sources (as was the case for run one), but the number of sources varied across individuals. Runs one through three assumed no correlation between the doses of each source. Runs four through six assumed an *m* of 100 and that all individuals were exposed, but the individuals’ values of *TD_jk_* of each source were rank correlated to varying degrees (Spearman correlation coefficients of 0.2, 0.5, and 0.8).

**Table I:** Inputs to the Six Model Runs

Runs	Number of Sources (m)	α_k‐min_	α_k‐max_	*PS_GM_*	*PM_GSD_*	Spearman Correlation Coefficient
1	20	1	1	6	2.5	0
2	100	1	1	6	2.5	0
3	100	0.2	0.2	6	2.5	0
4	100	1	1	6	2.5	0.2
5	100	1	1	6	2.5	0.5
6	100	1	1	6	2.5	0.8

All the model runs varied *GM_k_*, $GSD_{k}$ using the approach described above. In all six runs, the values of *PS_GM_* and *PM_GSD_* were set at 6.0 and 2.5. A value of 6.0 for *PS_GM_* indicates that the values of *GM_k_* for the sources of some population could be very similar in size and in other populations could vary by a factor of 1 million. A value of 2.5 for *PM_GSD_* indicates that the sources of some populations provide a uniform dose across all individuals (*M_GSD_* equal to 0). For populations where *M_GSD_* has a value of 2.5, the log_10_(*GSD_k_*)*_max_* of a source could be as large as five. A source with a log_10_(*GSD_k_*) of five would have *TD_jk_* values at plus or minus two geometric standard deviations of the geometric mean that would differ by more than 20 orders of magnitude.

The results of each model run were used to generate a series of six scatter plots that show the effect of the spread of the average range of *GM_k_* and the average value of *GSD_k_*.in a population on the three population metrics. These two parameters were selected for the following reasons. First, the impacts of variation in values of the other model parameters (e.g., *m* and *α*) are explored in the different model runs. Second, as discussed above, variation in the average of the GM for a population's sources has no impact on the population metrics. Finally, while both the average and the spread of GSD values across a population's sources affect the metrics, the populations’ spread of the GSD values are tightly correlated to the populations’ average GSD values. As a result, plotting the three metrics against the spread of GSD gives very similar plots to the plots against average GSD (results not shown).

## 3 RESULTS

**Fig 3:**
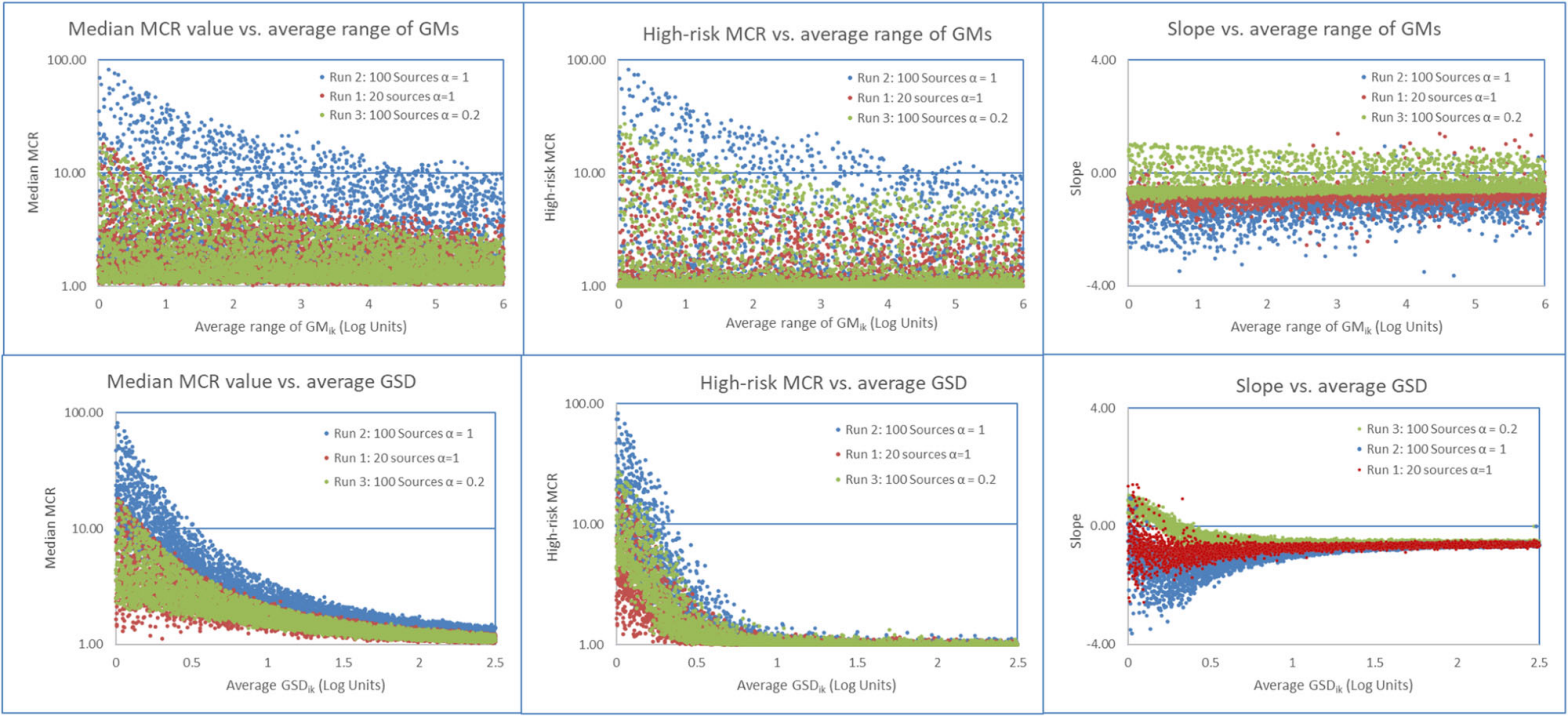
Effect of increasing the average range of geometric means and average geometric standard deviation of the source‐specific toxicity‐weighted doses on the three population metrics in runs one to three.

**Fig 4:**
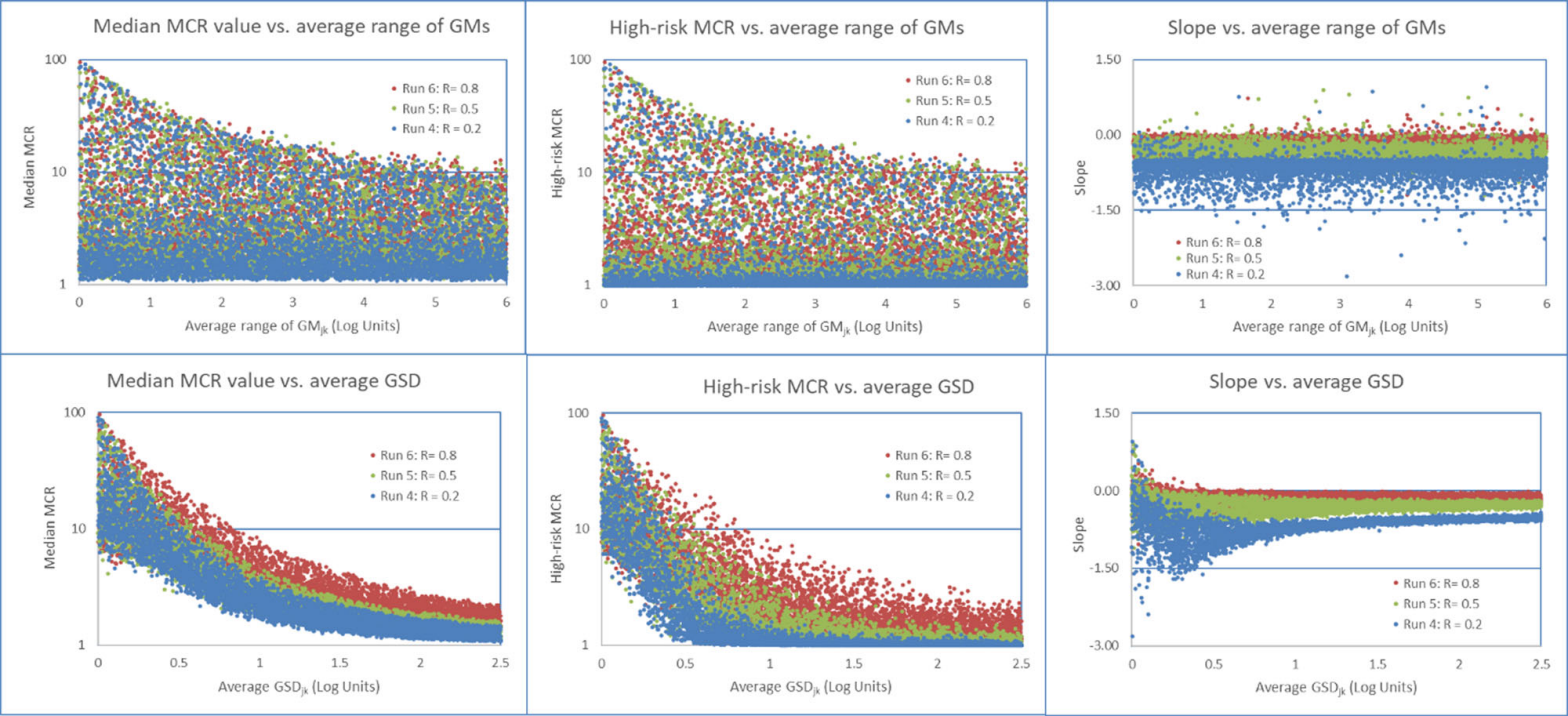
Impact of different degrees of rank correlation on the relationships between the average range of geometric means and average geometric standard deviations of the distributions of the source‐specific doses and the three population metrics in runs four to six.

The results for the six model runs are presented as a series of scatter plots and a summary table. Fig. 3 shows the results for model runs one through three, and Fig. 4 shows the results for runs four through six. Each figure presents the six scatter plots of the three population metrics (median MCR, high‐risk MCR, and MCR slope) plotted against the average of the range of *GM_k_* and the *GSD* *_k_* of the distributions of populations’ doses.

Table II gives information on the values of the three population metrics for the 5,000 populations simulated in each of the six model runs. Results are given for all populations in a run and for populations with average GSD values above and below 0.5. To capture the range of values for the metrics across the 5,000 populations, three percentiles (2.5, 50, and 97.5) are reported. The range of values of the metrics can be viewed as the range of plausible values that could occur when certain criteria are defined for a population.

**Table II:** Values of the Three Population Metrics in Runs Four to Six

		Median MCR			High‐Risk MCR			Slope		
	% Pop.	All Pop.	Pop. with Avg. *GSD_k_* <0.5	Pop. with Avg. *GSD_k_* >0.5	All Pop.	Pop. with Avg. *GSD_k_* <0.5	Pop. with Avg. *GSD_k_* >0.5	All Pop.	Pop. with Avg. *GSD_k_* <0.5	Pop. with Avg. *GSD_k_* >0.5
Run 1: 20 sources, all exposed	2.5	1.1	1.6	1.1	1	1.1	1	−1.3	−1.6	−0.95
	50	1.5	3.3	1.4	1	1.9	1	−0.67	−0.86	−0.66
	97.5	6.4	11	2.8	4.5	10	1.2	−0.47	0.34	−0.55
Run 2: 100 sources, all exposed	2.5	1.1	3.9	1.1	1	1.2	1	−2.1	−2.6	−1.4
	50	1.9	8.9	1.6	1	4.9	1	−0.76	−1.4	−0.71
	97.5	21	43	5	17	38	1.6	−0.58	−0.34	−0.59
Run 3: 100 sources, 20% exposed	2.5	1.1	2	1.1	1	1.1	1	−0.77	−0.76	−0.77
	50	1.5	3.2	1.4	1	2.9	1	−0.57	0.06	−0.59
	97.5	6.2	10.1	2.8	7.4	14.4	1.3	0.63	0.9	−0.39
Run 4: Rank correlation = 0.2	2.5	1.1	4.1	1.1	1	1.3	1	−1.2	−1.5	−0.97
	50	1.5	3.2	1.4	1	2.9	1	−0.57	0.06	−0.59
	97.5	22	47	5.1	20	43	2.4	−0.41	−0.07	−0.49
Run 5: Rank correlation = 0.5	2.5	1.2	4.4	1.2	1	1.4	1	−0.61	−0.75	−0.52
	50	2.1	9.5	1.8	1.2	7.7	1.1	−0.3	−0.28	−0.3
	97.5	22	48	6	21	48	4.7	−0.074	0.079	−0.18
Run 5: Rank correlation = 0.8	2.5	1.5	5.4	1.5	1	2.5	1	−0.36	−0.46	−0.33
	50	3.2	11	2.6	2.1	9.5	1.6	−0.15	−0.11	−0.15
	97.5	27	50	9.7	24	45	8.1	0.0035	0.25	−0.04

The size of the ranges of *GM_k_* values for a population's sources has a modest effect on *MCR_j_*, with wider ranges of *GM_k_* suppressing *MCR_j_* values. The size had little effect on the MCR slopes. When the ranges of *GM_k_* values for a population's sources are small, median MCR values approach *m* for a small number of populations.

Differences in the average *GSD_k_* of the source‐specific doses have a larger effect on *MCR_j_* and the MCR slope than the average range of the *GM_k_*. Values of *MCR_j_* only approach *m* when values of average *GSD_k_* are close to zero. Populations where the average *GSD_k_* value is greater than 0.5 (individuals at two standard deviations above and below the GM differ by a factor of 100 or greater) have low values of *MCR_j_*, negative slopes, and values of *MCR_j_* are largely independent of *m* and $\alpha_{k}$. In populations with average *GSD_k_* values of less than 0.5, the values of *MCR_j_* become dependent on *m* and $\alpha_{k}$. Positive MCR slopes in runs one and two increase with *m* but positive slopes are limited to the small fraction of the populations where the average values of *GSD_k_* are less than 0.2.

In the first three runs, the majority of the populations display the pattern of small values (relative to *m*) for the median and high‐risk MCR and negative MCR slopes (Fig. 3). The fivefold increase in *m* between runs one and two increases the various measures of *MCR_j_* values. The size of the increase varies for different percentiles of median and high‐risk MCR (Table II); but all increases are smaller than the increase in *m*. The 2.5 and 50th percentiles of the median and high‐risk MCR are largely unchanged; but, the 97.5th percentiles of the two metrics increased by factors of 3.2 and 3.8, respectively. As stated above, the effects of an increase in *m* on MCR are more pronounced when *GSD_k_* values are less than 0.5. Increasing *m* made the MCR slopes more negative.

In run three, where the number of sources that reach an individual varies across individuals, the fraction of the MCR slopes that are positive increases compared to run one where the number is constant (Fig. 3). The values of the three percentiles of median MCR are largely unchanged between run one and three, but the values 50th and 97.5th percentiles of the high‐risk MCR increase by roughly a factor of 1.5 (Table II). Again, the differences between the two runs are most pronounced when *GSD_k_* values are less than 0.5.

Runs four through six investigate the impact of introducing correlations into an individual's *TD_ijk_* values (Fig. 4). While the pattern observed in the first three runs occurs in these runs as well, correlations in source‐specific values reduce the interindividual variation in an individual's *TD_ijk_* values and as a result, values of median and high‐risk MCR increase and the MCR slopes become flatter with increasing correlation. The impacts are most significant for populations where all correlations coefficients are greater than 0.2 and average *GSD_k_* values are less than 0.5.

## 4 DISCUSSION

This article began with the observation that a “pattern” is frequently observed in studies of combined exposures. In this pattern, risks posed by such combined exposures are almost always driven by exposures from a few chemicals and sources and the exposures to individuals with the highest combined exposures are frequently driven by a single chemical from a single source. This analysis used computer simulations of combined exposures to investigate when multiple sources of chemicals drive the largest risks in a population and when a chemical from a single source is responsible for the largest risks. The modeling demonstrated that the “pattern” consistenly occurres when distributions of source‐specific doses follow right‐skewed distributions with the following exceptions. As shown in Fig. 3 (runs one through three) and in Table II, the pattern does not occur in simulated populations where the interindividual variation of source‐specific dosesare small. Fig. 4 (runs four through six) shows that moderate‐to‐high correlations between source‐specific doses increase the likelihood that the pattern would not occur. Finally, variation in the number of sources affecting different individuals in the population and similar measures of central tendency across source‐specific doses further suppress the occurance of the pattern when the interindividual variation of source‐specific doses are small (Fig. 3 and Table II).

As discussed in the introductin, the finding that this pattern widely occurs has implications for assessments of risks from combined exposures. Values of the high‐risk MCR close to one and findings of safety for each source of exposure implies that the risk from combined exposures will also be safe. In such cases, there may be no need to determine a population's combined exposures. This is a valuable finding since combined exposure assessments are often difficult to perform and can be resource intensive.

The problem with using high‐risk MCR values in determining the need for assessing combined exposures is that the values MCR for a population cannot be determined unless a combined exposure assessment has already been performed (Organisation for Economic Co‐operation and Development, 2018). This limits the use of MCR to retroactive analyses. This article presents a solution to this problem by demonstrating relationships between measurable characteristics of source‐specific doses in exposed populations that could be used to predict when the “pattern” occurs and high‐risk MCR values will be close to one. Specifically, small values of high‐risk MCR occur in populations where the distributions of sources‐specific doses have average GSDs that are greater than 0.5, have GMs of different sizes, the number of sources‐specific doses that reach an individual are constant over the population, and there are low or no correlations between individuals’ source‐specific doses.

With additional research, it may be possible to develop criteria, or processes, that allow data on interindividual variation to quantitatively predict if risks from combined exposures exceed the risks that occur from separate exposures to individual chemicals and sources. Under such approaches, data would be collected on the number of sources affecting a common population, correlations between the source‐specific doses, and the size of the variation in the doses across individuals. Based on such data, estimates would be made on the upper bound of the likely range of the values for the population's high‐risk MCR. This upper bound value could be multiplied times the largest upper bound estimate of the source‐specific doses for the population. If the resulting estimate of exposure is acceptable, then the assessor would have a basis for concluding that combined risks do not pose a problem for the population.

The use of a multiplicative factor to account for combined exposures has been suggested in the past (KEMI, 2015; Martin, Martin, & Kortenkamp, 2013) but researchers have struggled to develop objective criteria to determine the size of such a factor. The concept of the high‐risk MCR and the relationship between the high‐risk MCR and the characteristics of distributions of source‐specific doses established in this article may be helpful in such endeavors.

Finally, the initial part of the “pattern” (that the sum of separate values is often driven by a small number of the values) is not a novel finding. It has been found to occur in many fields (the majority of sales tend to come from a minority of clients, the majority of charitable donations come from a few contributors, and the majority of one's Facebook posts come from a small number of Facebook friends). The early observation of this behavior was attributed to Vilfredo Pareto and is frequently termed the Pareto principle (Backhaus, 1980; Juran, 2005). The second portion of the pattern (that the proportion of the sum from the largest value increases with the size of the sum for sums of values generated by sampling right‐skewed distributions) is believed to be a novel finding.

**NOMENCLATURE**
*AD_ij_*: aggregate dose of the *i*th chemical to the *j*th individual
*CTD_j_*: measure of combined toxicity adjusted doses for the *j*th individual
*D_ijk_*: dose of the *i*th chemical for the *j*th individual from the *k*th source
*GM_k_*: geometric mean of the nonzero portion of the distribution of $TD_{ijk}$received by individuals in a population from the *k*th source of exposure
*GSD_k_*: geometric standard deviation of the nonzero portion of the distribution of *TD_ijk_* received by individuals in a population from the *k*th source of exposure
*HQ_ijk_*: hazard quotient for the dose of the *i*th chemical for the *j*th individual from the *k*th source
HI: hazard index
*HI_j_*: hazard index for the *j*th individual
High‐risk MCR: mean value of MCR in the individuals with the 10 largest values of CTD of a population
ID: index chemical
*ID_ijk_*: dose of the index chemical that is equivalent to *D_ijk_*
*ID_j_*: total dose of the index chemical reaching the *j*th individual
*K_i_*: a weighting factor that converts a dose of the *i*th chemical to a metric (toxicity‐weighed dose) that can be summed and compared to a value of 1
*Max(TD_ij_)*: largest aggregate toxicity‐weighted dose of the *i*th chemical for the *j*th individual
MCR: maximum cumulative ratio
*MCR_j_*: value of MCR for the *j*th individual
*M_GSD_*: mean value of log_10_(*GSD_jk_*) across all sources of exposure for a population
Median MCR: median value of *MCR_j_* in a population
MCR slope: slope of a linear regression model of the log_10_(*MCR_j_* − 1) versus the log_10_(*CTG_j_*) for individuals in a population
*PS_GM_*: upper bound of *S_GM_* in the simulated populations in a model run
*PM_GSD_*: upper bound of *M_GSD_* in the simulated populations in a model run
*PS_GSD_*: upper bound of *S_GSD_* in a simulated population
*PD_i_*: permitted dose of the *i*th chemical
*PD_ID_*: permitted dose of the index chemical
*S_GM_*: spread parameter for log_10_(*GM_k_*)
*S_GSD_*: spread parameter for log_10_(*GSD_k_*)
*TD_ijk_*: toxicity adjusted dose of the *i*th chemical for the *j*th individual from the *k*th source.
*TEF_i_*: toxicity equivalent factor for the *i*th chemical
*TEq*: toxicity equivalent
*α_k_*: the portion of the population exposed to the *k*th source
$\hat{\alpha }$: a random number between 0 and 1

## Footnotes

1 Downloaded July 17/2019 from https://cfpub.epa.gov/ncea/iris/search/index.cfm
